# Molecular xenomonitoring highlights post-MDA surveillance priorities for sustained *Wuchereria bancrofti* elimination in Burkina Faso

**DOI:** 10.1186/s41182-025-00826-1

**Published:** 2025-10-30

**Authors:** Schawanya K. Rattanapitoon, Patpicha Arunsan, Chutharat Thanchonnang, Nathkapach K. Rattanapitoon

**Affiliations:** 1 Parasitic Disease Unit, FMC Medical Center, Nakhon Ratchasima, 30000 Thailand; 2 Faculty of Medicine, Vongchavalitkul University, Nakhon Ratchasima, Thailand; 3 Faculty of Public Health, Mahidol University, Nakhon Sawan Campus, Nakhon Sawan, Thailand

**Keywords:** *Wuchereria bancrofti*, Molecular xenomonitoring, Anopheles, Lymphatic filariasis elimination, Burkina Faso, One Health

## Abstract

The study by Nikièma et al. demonstrates the absence of *Wuchereria bancrofti* infection in Anopheles mosquitoes a decade after cessation of mass drug administration (MDA) in Burkina Faso. This rare longitudinal evidence underscores the utility of molecular xenomonitoring (MX) as a sensitive early-warning tool that complements traditional transmission assessment surveys (TAS). MX enables detection of recrudescence earlier than human-based diagnostics, particularly in low-prevalence settings, and offers opportunities for integration with malaria vector surveillance. Ethical and operational challenges associated with human landing catches highlight the need for alternative trapping methods to maintain MX sensitivity safely. Sustaining lymphatic filariasis elimination globally will depend on institutionalizing MX alongside TAS, ensuring robust surveillance, and safeguarding long-term programmatic gains.

Dear Editor,

We commend the study by Nikièma et al. [1] for providing compelling evidence that *Wuchereria bancrofti* transmission remains undetectable in *Anopheles* mosquitoes a decade after mass drug administration (MDA) was halted in Burkina Faso. This rare longitudinal dataset is more than a reassurance for national elimination—it is a wake-up call for how surveillance must evolve in the “endgame” of lymphatic filariasis (LF).

First, the authors’ use of molecular xenomonitoring (MX) reinforces its value as a sensitive early-warning system. TAS, the cornerstone of WHO’s decision-making framework, has been criticized for limited sensitivity in detecting focal recrudescence, especially where prevalence has fallen to very low levels [2]. By directly testing vectors, MX bypasses the lag inherent in human antigen or microfilaria detection, offering a safeguard against premature cessation of interventions—a mistake documented in other LF programs [3].

Second, the study underscores the potential for programmatic integration. In West Africa, *Anopheles gambiae* sensu lato serves as both the principal malaria and LF vector. This overlap provides a unique opportunity: MX could be embedded within existing malaria entomological surveillance platforms. Such an integrated approach would not only be cost-efficient but would also embody the cross-disease synergy that One Health frameworks encourage [4].

Third, the reliance on human landing catches (HLC) raises ethical and logistical dilemmas. The findings of Nikièma et al. highlight the urgency of validating innovative trapping methods—such as CDC light traps, BG-Sentinel traps, or attractant-baited devices—that can standardize MX without relying on human exposure. Establishing such alternatives will be critical for scaling MX across endemic countries in Africa and Asia.

Looking forward, we believe that Burkina Faso’s experience should inform global LF policy. Institutionalizing MX as a routine complement to TAS could serve as a cornerstone for post-MDA surveillance strategies. By embedding MX within integrated vector platforms, national programs can build resilience against recrudescence and move closer to the 2030 elimination goals.

In conclusion, the study by Nikièma et al. provides not just local reassurance but also global direction: the path to sustaining LF elimination lies in embracing MX as a programmatic tool, moving beyond reliance on TAS alone, and ensuring that surveillance systems remain robust long after MDA has ceased.

## Data Availability

No datasets were generated or analysed during the current study.
